# Effects of Polymers on the Drug Solubility and Dissolution Enhancement of Poorly Water-Soluble Rivaroxaban

**DOI:** 10.3390/ijms23169491

**Published:** 2022-08-22

**Authors:** Min-Jong Choi, Mi Ran Woo, Han-Gon Choi, Sung Giu Jin

**Affiliations:** 1 Department of Pharmaceutical Engineering, Dankook University, 119 Dandae-ro, Dongnam-gu, Cheonan 31116, Korea; 2 College of Pharmacy, Hanyang University, 55 Hanyangdaehak-ro, Sangnok-gu, Ansan 15588, Korea

**Keywords:** rivaroxaban, polymeric solid dispersion, solubility, dissolution, physicochemical property

## Abstract

The purpose of this study was to investigate the efficacy of hydrophilic polymers in a solid dispersion formulation in improving the solubility and dissolution rate of rivaroxaban (RXB), a poorly soluble drug. The developed solid dispersion consisted of two components, a drug and a polymer, and the drug was dispersed as amorphous particles in a polymer matrix using the spray drying method. Polymeric solid dispersions were evaluated using solubility tests, in vitro dissolution tests, powder X-ray diffraction, differential scanning calorimetry, scanning electron microscopy, and particle size distribution analysis. To maximize physical stability against crystallization and improve the solubility and dissolution of RXB, it is important to select the appropriate polymer type and the optimal ratio of the polymer to the drug. The optimized polyvinyl alcohol (PVA)-based (1/0.5, *w*/*w*) and gelatin-based (1/5, *w*/*w*) solid dispersion formulations showed 6.3 and 3.6 times higher drug solubilities than pure RXB powder, respectively, and the final dissolution rate was improved by approximately 1.5 times. Scanning electron microscopy and particle size distribution analyses confirmed that the gelatin-based solid dispersion was smaller and more spherical than the PVA-based solid dispersion, suggesting that the gelatin-based solid dispersion had a faster initial dissolution rate. Differential scanning calorimetry and powder X-ray diffraction analyses confirmed that RXB had successfully changed from a crystalline form to an amorphous form, contributing to the improvement in its solubility and dissolution rate. This study provides a strategy for selecting suitable polymers for the development of amorphous polymer solid dispersions that can overcome precipitation during dissolution and stabilization of the amorphous state. In addition, the selected polymer solid dispersion improved the drug solubility and dissolution rate of RXB, a poorly soluble drug, and may be used as a promising drug delivery system.

## 1. Introduction

Rivaroxaban (RXB; 5-Chloro-N-((2-oxo-3-(4-(3-oxomorpholino)phenyl)oxazolidin-5-yl)methyl)thiophene-2-carboxamide) is an orally active anticoagulant and direct factor Xa inhibitor used to treat and prevent deep venous thrombosis [1]. RXB is classified as a class II (Biopharmaceutics Classification System) drug with low aqueous solubility and high permeability [2]. To overcome the solubility of poorly soluble drugs, various solubilization techniques, including physical and chemical modification methods, such as solid dispersion, encapsulation in nanoparticles, salt formation, milling, hot melt extrusion, freeze-drying, nanosuspension, cyclodextrin complexes, co-crystals, microneedles, micelle solubilization, and self-emulsifying drug delivery systems, have been studied [3,4,5,6,7]. Among various technologies, the widely used industrial solid dispersion technology has the advantage of dispersing the drug in a carrier and converting the drug from crystalline to amorphous form to increase the solubility, reduce the particle size, and improve its wettability [8]. Amorphous solid dispersions have been widely used as effective formulations to improve the bioavailability of poorly soluble drugs [9]. Therefore, in this study, RXB, a poorly soluble drug, was converted from a crystalline to an amorphous form by dispersing the drug in a polymer using a solid dispersion technique. The amorphous form of the pharmaceutical formulation has significantly improved solubility and dissolution rate compared to the crystalline form, but the amorphous state is a high-energy state that is physically unstable, which causes stability problems [10]. The polymers used to overcome this problem have shown promise as crystallization inhibitors and have been used to ensure a prolonged supersaturated state of the active pharmaceutical ingredient [11]. The degree of increase in the solubility, dissolution rate, and bioavailability of poorly soluble drugs differs depending on the polymer material used as the carrier of the solid dispersion, and the degree of inhibition of crystallization in the amorphous state of poorly soluble drugs also depends on the polymer. Therefore, the selection of a polymer for solid dispersion is important for the solubilization of poorly soluble drugs. In this study, the degree of solubility and dissolution of poorly soluble drugs was compared according to the properties of polymers. By comparing the characteristics of RXB according to the type and ratio of the polymer, it was attempted to improve the understanding of the change in the characteristics of RXB.

Polyvinyl alcohol (PVA), the polymer selected in this study, can be used as a carrier in solid dispersions, and it helps to achieve high solubility and bioavailability by inducing rapid absorption by solubilizing poorly soluble drugs [12]. PVA is a non-ionic pH-independent, water-soluble, safe, and biocompatible polymer that exhibits the same solubilizing performance throughout the gastrointestinal tract; hence, it is used as a polymer, leading to low variability and high bioavailability [13,14]. In addition, as a hydrophilic polymer, gelatin has the advantages of good biocompatibility, solubility, inertness, and easy acquisition, and it is a suitable polymer for the microencapsulation of poorly soluble drugs via spray drying to enhance its aqueous solubility and oral bioavailability [15]. Gelatin is a US Food and Drug Administration-approved natural polymer that is low cost and shows excellent biocompatibility and biodegradability [16]. There have been studies on the development of a solid dispersion by freeze-drying gelatin as a carrier [17], and studies on encapsulating curcumin in gelatin by electro-spray drying [18].

In this study, various solid dispersion formulations were prepared by spray drying hydrophilic polymers to develop RXB-supported solid dispersions with improved solubilities and dissolution rates. In addition to appropriate polymer selection, the drug–polymer ratio is an important factor that can affect the physical stability of RXB. The solubility, dissolution rate, thermal properties, and crystallinity of the solid dispersion were investigated according to the type and amount of the hydrophilic polymer.

Hence, the essential objective of our study is to develop and evaluate novel RXB solid dispersions with spray drying technology to improve solubility and dissolution of poorly soluble drugs. The solid dispersions of RXB using spray drying technology and particle size reduction according to the type of polymer were used in combination. We hypothesize that solid dispersion of the drug will add to the degree of solubility improvement depending on the type of polymer used and the drug/polymer ratio. The physicochemical properties of the solid dispersion and the evaluation of the solubility/dissolution behavior of RXB were further emphasized.

## 2. Results and Discussion

### 2.1. Effect of Polymer Concentration on the Solubility of RXB

**Figure 1:**
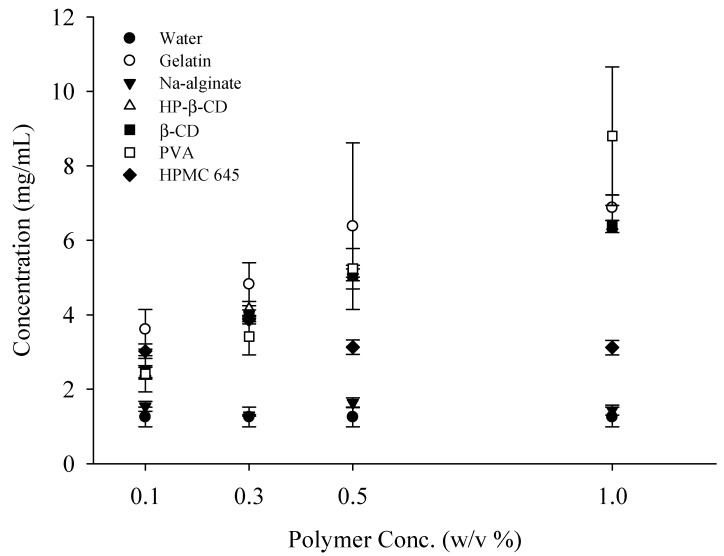
Effect of polymer concentration (0.1, 0.3, 0.5, and 1%) on the solubility of RXB. Each value shows the mean ± standard deviation (S.D.) (*n* = 3).

To select suitable hydrophilic polymers as carriers for RXB-loaded solid dispersions, the solubility of RXB in aqueous solutions containing various concentrations of hydrophilic polymers was evaluated (Figure 1). Each polymer aqueous solution was used at concentrations of 0.1, 0.3, 0.5, and 1% (*w*/*v*). The aqueous solubility of RXB is approximately 1.25 μg/mL, indicating that it is a poorly soluble drug. Polymer selection is very important to increase the solubility of poorly soluble drugs. The evaluation of drug solubility depending on the polymer to select an appropriate polymer is usually performed at one concentration [15]. However, the increase in solubility of a drug according to the concentration of the polymer depends on the type of polymer [19]. In general, the solubility of poorly soluble drugs increases with the amount of polymer in the solid dispersion, but in this study, the rate of solubility increase of the drug was different depending on the type of polymer. The evaluation of the solubility of RXB at various hydrophilic polymer concentrations showed that PVA improved drug solubility the most compared to other polymers at high concentrations (1 *w*/*v*%). Meanwhile, gelatin enhanced the drug solubility compared to other polymers at low concentrations (0.1–0.5 *w*/*v*%). However, Na-alginate showed little increase in solubility with an increasing polymer concentration. HPMC 645 decreased drug solubility with the increasing polymer concentration. These results suggest that the choice of polymer is a very important factor in increasing the solubility of poorly soluble RXB. Therefore, PVA and gelatin were chosen as carriers for the development of solid dispersions loaded with RXB.

### 2.2. Effect of Polymer Concentration on the Solubility of RXB-Loaded Solid Dispersions

**Figure 2:**
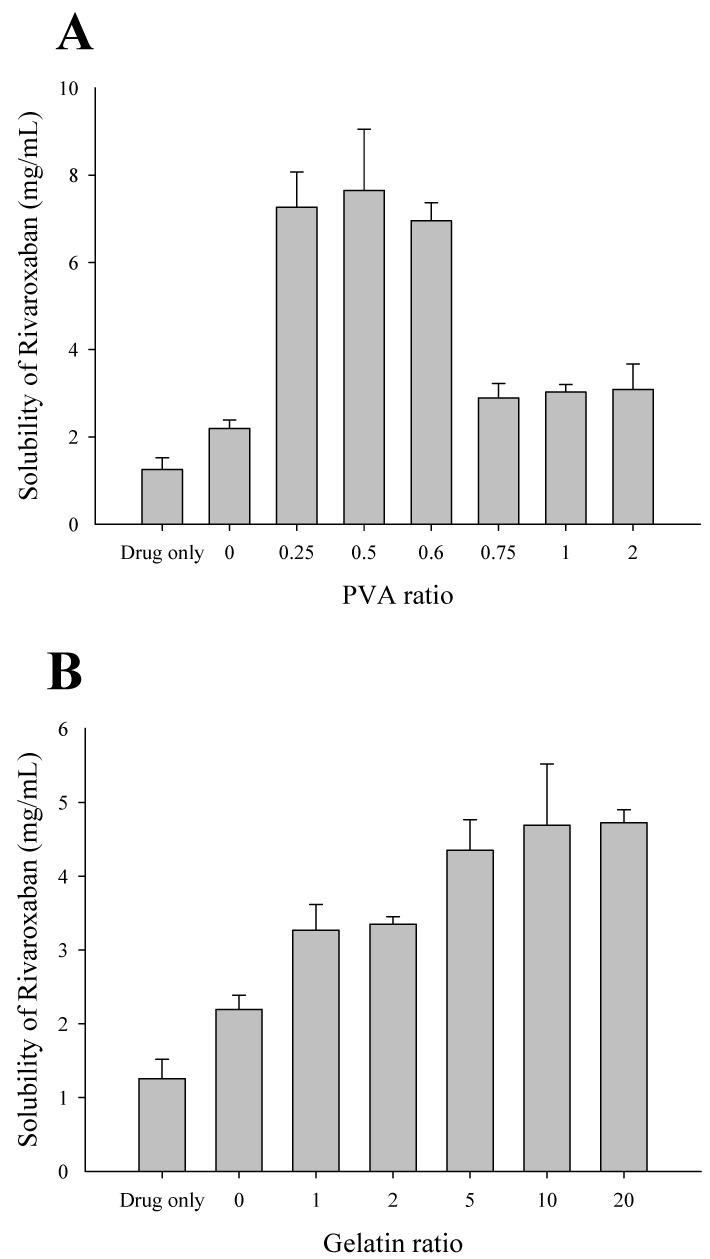
Effect of polymer concentration on the solubility of RXB-loaded solid dispersions. (**A**) Polyvinyl alcohol (PVA). (**B**) Gelatin. Each value shows the mean ± S.D. (*n* = 3).

The effect of the drug/polymer ratio on drug solubility was investigated to select an appropriate ratio of the polymer for RXB-loaded solid dispersions (Figure 2). First, PVA-based solid dispersions were prepared with weight ratios of RXB to PVA of 1/0, 1/0.25, 1/0.5, 1/0.6, 1/0.75, 1/1, and 1/2 using the spray drying method. The effect of the RXB/PVA ratio on the aqueous solubility of the drug in the RXB-loaded solid dispersions was investigated (Figure 2A). As the amount of PVA was gradually increased, the solubility of RXB in the solid dispersion increased significantly. In general, the solubility of poorly soluble drugs tends to increase with the number of water-soluble polymers in the solid dispersion [20,21]. However, after the RXB/PVA ratio reached 1/0.5, the solubility decreased as the amount of polymer increased. This result indicates that the ratio of the drug to the polymer as the type of polymer in the solid dispersion is also an important factor in increasing the drug solubility. Lee et al. (2021) showed that solubility decreased as the concentration of PVA increased compared to other polymers in solid dispersion studies using hot-melting extrusion. Therefore, it showed that the ratio of drug to polymer is important for increasing the solubility of RXB [19]. In addition, Liu et al. (2022) revealed that drug–polymer interactions are important for dissolution performance in solid dispersions. According to the ratio of felodipine to the polymer, the dissolution performance was dependent on the drug–polymer interaction [22]. Therefore, in this study, PVA-based solid dispersion with a weight ratio of 1:0.5 showed significantly higher solubility than other ratios and was selected for further study. Subsequently, gelatin-based solid dispersions were prepared with 1 g of RXB and 0, 1, 2, 5, 10, or 20 g of gelatin using the spray drying method. The influence of the amount of gelatin on the aqueous solubility of RXB in solid dispersions was evaluated (Figure 2B). Unlike PVA-based solid dispersions, the aqueous solubility of RXB increased as the ratio of gelatin among various gelatin solid dispersions increased, and there was no significant difference in the solubility when the gelatin content was ≥5 g. Similar to the solubility evaluation results of general solid dispersions, the solubility of solid dispersion did not decrease but increased as the polymer ratio increased [23]. As a result, we selected solid dispersions with RXB/gelatin ratios of 1/5 with high solubility and a minimal amount of carrier. The drug–polymer ratio had a different effect on improving the drug solubility depending on the type of polymer [24]. Depending on the polymer selection, the selected PVA and gelatin-based solid dispersions prepared via the spray drying method increased the drug solubility about 6- and 3.5-fold, respectively, compared to the drug powder. These results suggest that determining the optimal drug/polymer ratio based on the polymer type is important for maximizing the benefits of solid dispersion systems, as a higher amount of polymer does not always lead to higher drug solubility. It also suggests that other drug/polymer ratios using different polymers may contain limitations that may result in higher solubility.

### 2.3. Effect of Polymer Concentration on Dissolution Rate of RXB-Loaded Solid Dispersions

**Figure 3:**
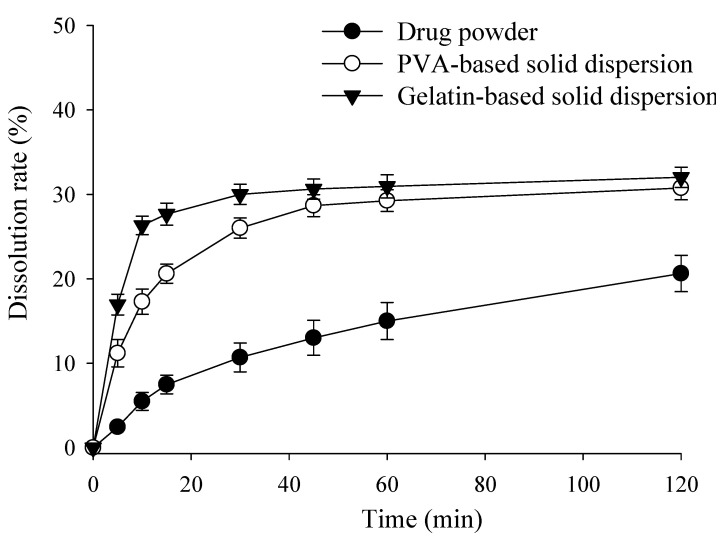
Effect of polymer concentration on the dissolution rate of RXB-loaded solid dispersions. Each value shows the mean ± S.D. (*n* = 3).

In vitro dissolution rates of RXB and the prepared PVA- and gelatin-based solid dispersions (Table 1) are shown in Figure 3. The dissolution rates of PVA- and gelatin-based solid dispersions were approximately 30.7 ± 1.4% and 32.1 ± 1.2%, respectively, at 120 min, indicating a significantly increased dissolution rate compared to the pure drug powder (20.6 ± 2.1%). This was due to the increased wettability and dispersibility of the drug with the preparation of the solid dispersion by the hydrophilic carrier. Hydrophilic carriers used to prepare solid dispersions contribute to improving the wettability and reducing the interfacial tension between the hydrophobic drug and the dissolution medium [25]. In addition, by evaluating the effect of the polymer on the dissolution of RXB by comparing the initial dissolution rates of PVA and gelatin-based solid dispersions, it was found that the dissolution rate of gelatin-based solid dispersions was significantly increased in the initial 30 min compared with PVA-based solid dispersions. This suggests that gelatin-based solid dispersion has higher initial wettability compared to PVA. However, the final dissolution rate showed similar results for PVA and gelatin-based solid dispersion. To increase patient convenience, the final formulation needs to be designed with a small amount, so it is necessary to use as little polymer as possible in the final formulation. The polymer amount of PVA-based solid dispersion was less than that of gelatin-based solid dispersion, but the final dissolution rate was similar. In the previous study by Metre et al. (2018), a study of a solid dispersion using Eudragit-type polymers was conducted, and the initial and final dissolution rates were different depending on the Eudragit type. This suggests that the appropriate polymer selection is important in increasing the dissolution rate of RXB [26]. Although the dissolution condition was a sink condition, the dissolution rate of the PVA and gelatin-based solid dispersion was stabilized after 60 min and showed about 30%. Since the dissolution evaluation indicates a short-term dissolution rate, it seems to indicate a limited final dissolution rate compared to the solubility result reflecting long-term saturated solubility [26,27].

### 2.4. Physicochemical Properties of RXB-Loaded Solid Dispersions

#### 2.4.1. Scanning Electron Microscopy (SEM) and Particle Size Distribution Studies

**Figure 4:**
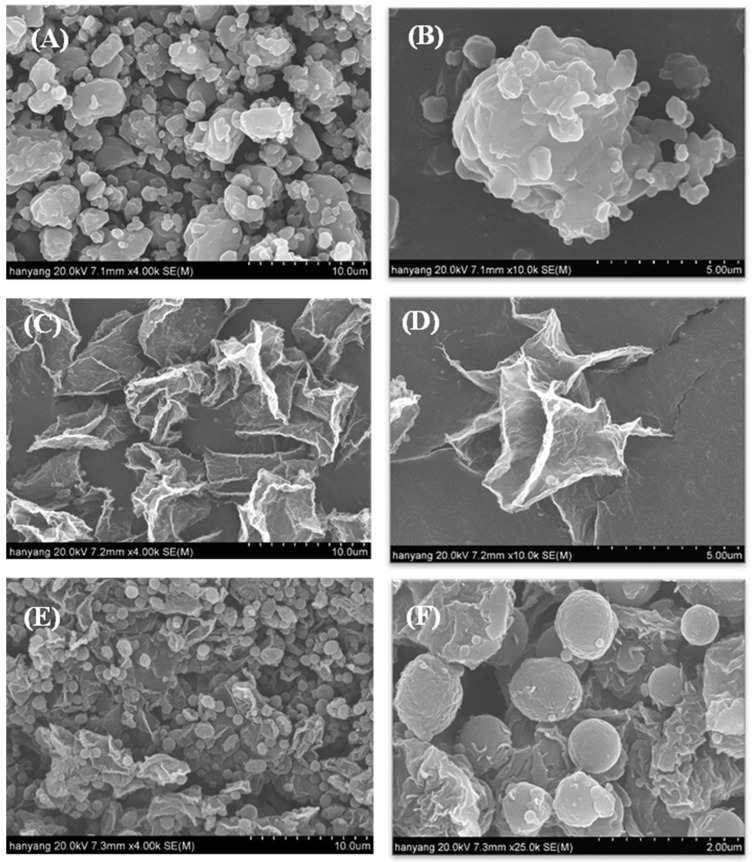
Scanning electron micrographs: (**A**) RXB powder (×4000); (**B**) RXB powder (×10,000); (**C**) PVA-based solid dispersions (×4000); (**D**) PVA-based solid dispersions (×10,000); (**E**) gelatin-based solid dispersions (×4000); and (**F**) gelatin-based solid dispersions (×25,000).

The surface morphology of the material was observed via SEM. SEM images of the RXB powder, PVA-based solid dispersions, and gelatin-based solid dispersions are shown in Figure 4. RXB powder (Figure 4A,B) has a smooth surface with a rectangular and irregular crystalline shape [25]. However, the solid dispersions did not contain any RXB crystals except large particles, suggesting that the drug and polymer were homogeneously combined in the solid dispersions (Figure 4C–F) [28]. PVA-based solid dispersions (Figure 4C,D) appeared as irregular and flaky particles with a low polymer ratio, whereas the gelatin-based solid dispersions (Figure 4E,F) were spherical with a smooth surface. The SEM images of gelatin-based solid dispersions showed smaller spherical particles than those of pure RXB powder and PVA-based solid dispersions. This demonstrated that the initial dissolution rate of gelatin-based solid dispersions is higher than that of PVA-based solid dispersions. These results also showed that the initial wettability of the gelatin-based solid dispersion assumed in the dissolution result was increased compared to the PVA-based solid dispersion. As the gelatin-based solid dispersion has a spherical shape and a smaller particle size than the PVA-based solid dispersion, the initial wettability was increased, which seems to have contributed to the improvement of the initial dissolution rate [21].

**Figure 5:**
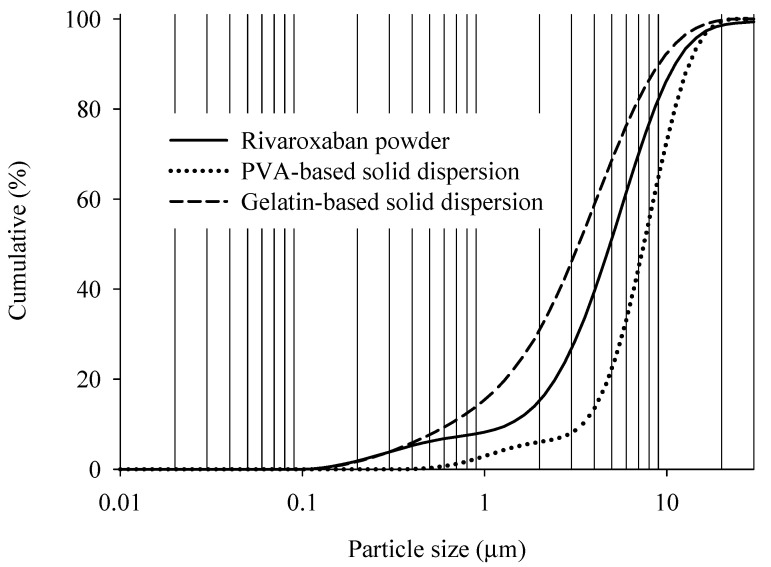
Particle size distribution of RXB and RXB-loaded solid dispersions.

A particle size analysis was performed, and the results are shown in Figure 5. The particle size affects the drug dissolution rate; therefore, it is important for solid dispersions to have a uniform particle size and shape [29]. The particle size of the gelatin-based solid dispersion was significantly reduced compared to the RXB powder in the cumulative particle size distribution. However, the particle size of the PVA-based solid dispersion did not decrease compared to the RXB powder in the cumulative particle size distribution, because the PVA-based solid dispersion particles showed an irregular flake structure with a wide particle size distribution. Our results suggest that the trends in the SEM results are consistent with the particle size distribution. There was a significant size reduction following morphological changes, especially in gelatin-based solid dispersion formulations [4]. These results showed that the increase in the wettability and the decrease in particle size of the gelatin-based solid dispersion were higher than the initial dissolution rate of the PVA-based solid dispersion.

#### 2.4.2. Differential Scanning Calorimetry (DSC) and Powder X-ray Diffraction (PXRD)

**Figure 6:**
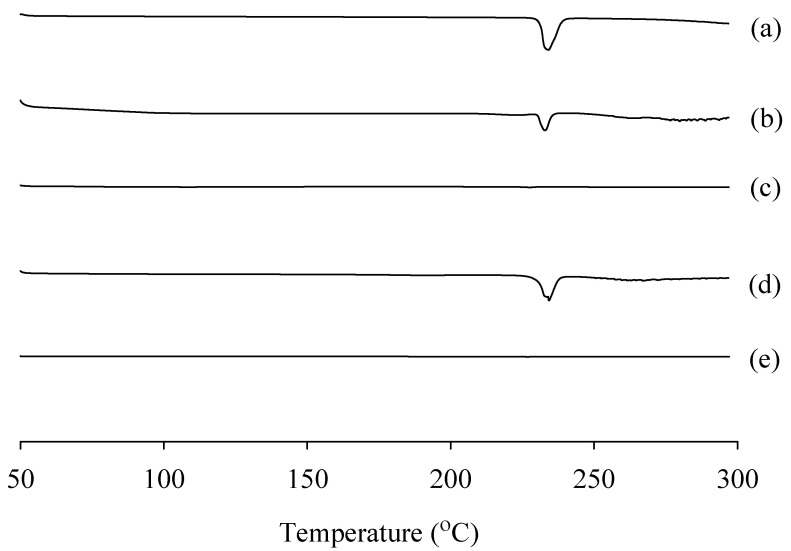
Comparison of differential scanning calorimetric thermograms: (a) RXB powder; (b) physical mixture of RXB and gelatin (1:5); (c) gelatin-based solid dispersions (1:5); (d) physical mixture of RXB and PVA (1:0.5); (e) PVA-based solid dispersions (1:0.5).

DSC was used to verify the thermal behavior of drugs in the formulation (Figure 6). The DSC curve of RXB powder (a) and physical mixtures (b, d) showed a sharp single endothermic peak appeared at about 228 °C, which corresponds to the melting point of RXB indicative of crystalline properties [28]. However, in the PVA-based and gelatin-based solid dispersions (c, e), the sharp characteristic peak corresponding to the melting point of the drug disappeared and the peak was not observed, indicating that the drug existed in an amorphous form [27]. The miscibility of the drug–polymer product manufactured through spray drying did not show any peak, unlike the physical mixture, indicating that RXB is uniformly distributed in the polymer matrix in a completely amorphous state [27].

**Figure 7:**
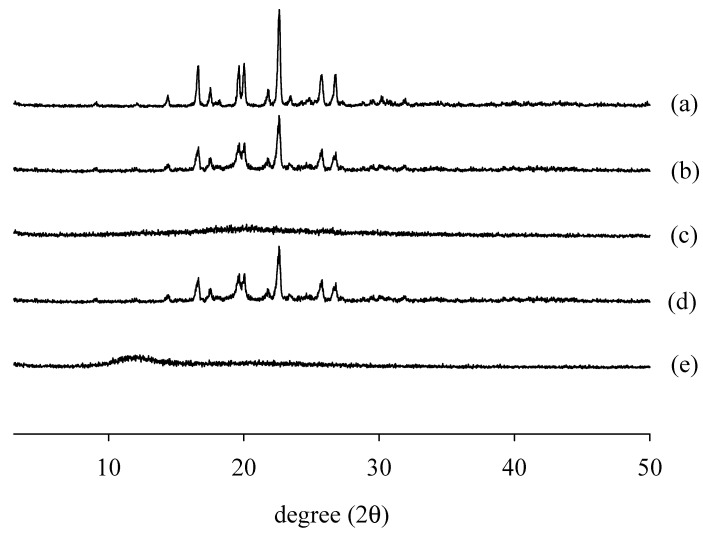
Comparison of Powder X-ray diffraction: (a) RXB powder; (b) physical mixture of RXB and gelatin (1:5); (c) gelatin-based solid dispersions (1:5); (d) physical mixture of RXB and PVA (1:0.5); (e) PVA-based solid dispersions (1:0.5).

PXRD was performed to confirm the crystallinity of RXB in the solid dispersions (Figure 7). The diffraction pattern of pure RXB (a) shows the maximum intensity at the 2θ value of 22 [4]. The diffractograms of the physical mixtures (b, d) showed distinct characteristic peaks of RXB, suggesting the presence of crystalline RXB. The decreased peak intensity with a slight shift/disappearance of the RXB peak in the physical mixture may be due to the dilution of the drug with the polymer. On the other hand, the diffraction patterns of the PVA-based and the gelatin-based solid dispersion showed a halo pattern without sharp intense peaks, suggesting that the crystalline form of the drug was changed to an amorphous form [30]. The change in crystallinity depending on the polymer in the solid dispersion was an important factor in improving the dissolution rate and solubility [31]. These physicochemical results showed that drug solubility was improved by changing the crystalline drug to an amorphous state via the spray drying method [32]. PVA and gelatin, used as carriers for solid dispersions, are expected to effectively increase the solubility and dissolution rates and increase the bioavailability of drugs, which is important to inhibit the crystallization of poorly soluble drugs in the amorphous state [33,34].

Therefore, the selected polymer-based solid dispersion improved the solubility and dissolution rate of poorly water-soluble RXB through changes in crystallinity compared to drug powder.

## 3. Materials and Methods

### 3.1. Materials

RXB was acquired from Hanmi Pharm. Co. (Suwon, Korea). HPMC 645 (4.5 cps) and PVA were purchased from Shin-Etsu. Co. (Tokyo, Japan). Gelatin and sodium alginate (Na-alginate) were obtained from Daejung Chemical Co. (Siheung, Korea). Hydroxypropyl-β-cyclodextrin (HP-β-CD) and β-cyclodextrin (β-CD) were purchased from Roquette (Lestrem, France). All other organic solvents used were of high-performance liquid chromatography (HPLC) grade.

### 3.2. Preparation of RXB-Loaded Spray-Dried Solid Dispersions

A solid dispersion of RXB was prepared using the spray drying method to determine the optimal carrier. PVA-based solid dispersions were spray-dried using a laboratory-scale Büchi nozzle-type mini spray dryer at an inlet temperature of 140 °C and an outlet temperature of 80–90 °C. The aspiration rate of the solution was set to 100% and the spray pressure was 4 kg/cm^2^. After dissolving RXB and various amounts of PVA in 2.5 L of ACN and distilled water (1:1.5, *v*/*v*), the solution was sprayed through a 0.7 mm pneumatic nozzle using a peristaltic pump at a flow rate of 10% [35]. In addition, gelatin-based solid dispersions were spray-dried at an inlet temperature of 105 °C and an outlet temperature of 60–70 °C. A feed solution consisting of RXB and various amounts of gelatin was dissolved in 6 L of ethanol and distilled water (1:1, *v*/*v*) and spray-dried [15].

### 3.3. Effect of Polymer on the Solubility of RXB

To select a suitable water-soluble polymer for the preparation of solid dispersions, the water solubility of RXB was screened in aqueous solutions of various hydrophilic polymers, such as gelatin, Na-alginate, HP-β-CD, β-CD, PVA, and HPMC 645. Each polymer aqueous solution was used at concentrations of 0.1, 0.3, 0.5, and 1% (*w*/*v*). Excess RXB was added to a 1 mL aqueous solution of each polymer, and the mixture was vortexed for 10 min and shaken in a water bath (Daihan Scientific, Wonju, Korea) at 25 °C for seven days. After centrifugation at 10,000× *g* for 10 min to obtain the supernatant, it was filtered through a 0.45 μm nylon filter [36]. Then, the filtrate was injected into the HPLC system to quantify the RXB concentration.

### 3.4. Solubility and Dissolution Study of RXB-Loaded Solid Dispersions

RXB-loaded solid dispersion samples (equivalent to 1 mg of RXB) were placed in a 2 mL microtube containing 1 mL of distilled water. The samples were vortexed at room temperature for 10 min and shaken for 3 d at 25 °C in a shaking water bath to achieve equilibrium [36]. The supernatant solution was passed through a 0.45 μm nylon filter and analyzed by HPLC after suitable dilution. All solubility studies were performed in triplicate.

Dissolution studies were performed according to the USP apparatus II paddle method using a dissolution tester (Vision Classic 6; Hanson Research Co., Chatsworth, CA, USA). Pure RXB powder, PVA-based solid dispersions, and gelatin-based solid dispersions, each containing 10 mg of RXB, were evaluated for dissolution rate in 900 mL of distilled water at 37 ± 0.5 °C with a rotation speed of 50 rpm [37]. Aliquot samples (3 mL) were withdrawn at specific time intervals (5, 10, 15, 30, 45, 60, and 120 min), filtered through a 0.45 μm nylon filter (Grade 1), and assayed for drug concentration by HPLC.

### 3.5. HPLC

HPLC analysis was conducted using an Agilent 1260 HPLC system (Agilent Technologies, Palo Alto, CA, USA) equipped with a degasser, pump, autosampler, and PDA detector. The solubility and dissolution tests were analyzed using a Shiseido Capcell Pak MG (4.6 × 150 mm, 5 μm) column with a mobile phase containing acetonitrile and 20 mM of ammonium acetate buffer solution adjusted to pH 4.3 at a ratio of 65:35 (*v*/*v*) at a flow rate of 1 mL/min. The temperature of the column was set at 35 °C, and the injection volume was 20 μL [2]. The drug concentration was determined based on a linear concentration range of 0.9–100.0 μg/mL (r^2^ = 0.9999) for the generated peak area at 250 nm. Each sample was analyzed in triplicate.

### 3.6. Physicochemical Properties of RXB-Loaded Solid Dispersions

#### 3.6.1. SEM Studies

The surface characteristics of the powder and solid dispersion samples were studied using SEM (S-4800; Hitachi, Tokyo, Japan). All samples were fixed to stubs using double-sided adhesive tape. The aluminum stubs were placed in a scanning electron microscope after vacuum coating with platinum using a sputter coating machine (EMI Tech K575K; Quorum Technologies Ltd., West Sussex, UK) and the surface morphological characterization was observed [38].

#### 3.6.2. DSC Study

DSC (DSC Q200; TA Instruments, New Castle, DE, USA) was used to study the thermal properties of the samples upon heating at a rate of 10 °C/min from 50 to 300 °C under a nitrogen gas flow. Approximately 3–5 mg of each sample was placed in an aluminum pan and measured with an empty pan as a reference [39].

#### 3.6.3. PXRD

PXRD was performed using a diffractometer (D/MAX-2500; Rigaku, Tokyo, Japan) with monochromatic Cu Kα radiation (γ = 1.54178 Å) [33]. The X-ray generator settings were 40 kV and 100 mA. Each diffractogram was recorded from 3 to 50° (2θ) at a scanning speed of 0.02°/s [40].

#### 3.6.4. Particle Size Distribution

Particle size distribution was used to study the particle size of the sample using a Mastersizer 3000 laser diffraction analyzer (Malvern, Worcestershire, UK). All samples were measured at an air pressure of 1.5 bar, 50% feed rate, and 1.5 mm hopper gap. The particle size distribution was assessed and expressed as D10, D50, and D90, corresponding to 10, 50, and 90% of the particle size distribution, respectively [41].

## 4. Conclusions

In this study, a polymer-based solid dispersion with hydrophilic polymers, such as PVA and gelatin, for improving the solubility and dissolution of RXB was successfully formulated, as evidenced by the results of the solubility, dissolution, and physicochemical property evaluations. The solubility, dissolution rate, physical properties, and particle size of RXB, a poorly soluble drug, were affected by the type and ratio of the hydrophilic polymer. In particular, the selection of the appropriate drug–polymer ratio changed the formulation from crystalline to amorphous state, which increased the water affinity and solubility of RXB. The solubility studies showed that the selected PVA-based (7.6 ± 1.4 µg/mL) and gelatin-based (4.3 ± 0.4 µg/mL) solid dispersions had significantly higher solubility compared to pure RXB powder (1.2 ± 0.3 µg/mL). In vitro dissolution studies revealed that the final dissolution rates of PVA-based (30.7 ± 1.4%) and gelatin-based (32.1 ± 1.2%) solid dispersions were significantly higher than that of pure RXB powder (20.6 ± 2.1%) at 120 min. In addition, it was confirmed that the initial dissolution of the gelatin-based solid dispersion was faster than that of the PVA-based solid dispersion because the gelatin-based solid dispersion consisted of spherical and small-sized particles. Therefore, our results suggest that the selection of the type and ratio of polymer is an important and promising strategy to improve the solubility of poorly soluble drugs and ultimately increase their bioavailability.

## Figures and Tables

**Table 1:** Composition of rivaroxaban (RXB)-loaded solid dispersions.

Formulations	PVA-Based Solid Dispersions	Gelatin-Based Solid Dispersions
Rivaroxaban (g)	1	1
PVA (g)	0.5	-
Gelatin (g)	-	5
Acetonitrile (L)	1	
Ethanol (L)	-	3
Distilled water (L)	1.5	3
